# Stage-specific modulation of multinucleation, fusion, and resorption by the long non-coding RNA DLEU1 and miR-16 in human primary osteoclasts

**DOI:** 10.1038/s41419-024-06983-1

**Published:** 2024-10-11

**Authors:** Sara Reis Moura, Ana Beatriz Sousa, Jacob Bastholm Olesen, Mário Adolfo Barbosa, Kent Søe, Maria Inês Almeida

**Affiliations:** 1 i3S—Instituto de Investigação e Inovação em Saúde, Universidade do Porto, Porto, Portugal; 2 INEB—Instituto de Engenharia Biomédica, Universidade do Porto, Porto, Portugal; 3 ICBAS—Instituto de Ciências Biomédicas Abel Salazar, Universidade do Porto, Porto, Portugal; 4 Department of Pathology, Odense University Hospital, Odense, Denmark; 5 Clinical Cell Biology, Pathology Research Unit, Department of Clinical Research, University of Southern Denmark, Odense, Denmark; 6 Department of Molecular Medicine, University of Southern Denmark, Odense, Denmark

**Keywords:** Cell biology, Metabolic disorders, Non-coding RNAs, RNAi, Nucleic-acid therapeutics

## Abstract

Osteoclasts are the only cells able to resorb all the constituents of the bone matrix. While the modulation of osteoclast activity is well established for preventing bone-related diseases, there is an increasing demand for novel classes of anti-resorption agents. Herein, we investigated non-coding RNA molecules and proposed *DLEU1* and miR-16 as potential candidates for modulating osteoclast functions. *DLEU1* and miR-16 target cell fusion at both the early and late stages of osteoclastogenesis but operate through independent pathways. *DLEU1* silencing hinders the fusion process, leading to abrogation of the phagocytic cup fusion modality and a reduction in the fusion events between mononucleated precursors and multinucleated osteoclasts, while miR-16 influences monocyte-to-osteoclast differentiation, impairing osteoclasts formation but not the number of nuclei at early stages. On the other hand, using these non-coding RNAs to engineer mature osteoclasts has implications for bone resorption. Both *DLEU1* and miR-16 influence the speed of resorption in pit-forming osteoclasts, without affecting the resorbed area. However, the impact of increasing miR-16 levels extends more broadly, affecting trench-forming osteoclasts as well, leading to a reduction in their percentage, speed, and resorbed area. These findings offer potential new therapeutic targets to ameliorate bone destruction in skeletal diseases.

## Introduction

Osteoporosis is a consequence of skeletal deterioration characterized by alterations in tissue microarchitecture and quality loss, resulting in increased vulnerability to fractures [1]. This condition is more prevalent in individuals older than 50 years of age and is caused by a disruption in the delicate balance between bone formation and resorption [2, 3]. In osteoporotic patients, the resorptive activity of osteoclasts (OCs) surpasses the bone formation capacity and is a hallmark of this disease [4].

OC formation comprises several stages, including precursor recruitment, differentiation, fusion, and activation/maturation. Each stage is essential and co-dependent for the proper functioning of the subsequent steps and ultimately determines the resorptive capacity of the OC [5, 6]. Therefore, any impairment in the early stages will have a subsequent influence on the later stages. In fact, a positive correlation between the nucleation status and the bone resorptive activity of the OCs has already been established in vitro [7–9]. Interestingly, this association between hyperactivation of multinucleation and increased bone resorption by the OC is also observed in patients with Paget’s disease, leading to exacerbated bone resorption [10–15]. In addition, a recent study reported that the fusion potential of human OCs in vitro is correlated with age, menopausal status, and bone resorption levels in vivo [16]. These findings reinforce that the relationship between fusion capacity and resorption level may not only be observed in vitro but also in vivo. Consequently, the regulation of bone destruction can be achieved either through modulation of preceding stages or directly at the resorptive stage. Mature OCs can remove bone tissue through two distinct strategies with very contrasting characteristics [17, 18]. On one hand, there is the pit mode, in which intermittent, restricted, and less aggressive erosion is observed [17, 18]. On the other hand, the trench mode is characterized by more aggressive bone resorption due to longer periods of bone resorption, faster erosion rates, deeper cavities, and sensitivity to cathepsin K (CTSK) inhibition [17, 18]. In fact, the inhibition of CTSK has been shown to abolish trench formation while enhancing pit formation [19, 20]. Considering the different degrees of aggressiveness associated with each resorption mode and their association with bone resorption in vivo [7], it seems appropriate to assume their potential clinical relevance. Therefore, in addition to evaluating overall bone resorption activity, investigating the impact of non-coding RNAs (ncRNAs) on both resorption modes separately is also of interest.

In recent decades, several factors, both intrinsic and extrinsic, have been identified as key players in the cascade of events that culminate in OC maturation, activation, and activity. Among them are NFATc1 (Nuclear Factor Of Activated T Cells 1) [21–23], DC-STAMP (dendritic cell-specific transmembrane protein) [24–29], CD47 [30–35], syncytin-1 [30, 36], and e-cadherin [37, 38]. Recently, ncRNAs have received increased interest due to their abnormal expression profiles [39], frequent association with disorders (e.g. bone disorders) [40], and ability to modulate distinct cellular processes involved in bone remodeling [2, 41–45]. Nevertheless, a deep and extensive understanding of the role of specific microRNAs (miRNAs) and of long non-coding RNAs (lncRNAs) is needed for future clinical translation.

In this study, we investigated the role of small and long ncRNAs located at chromosomal region 13q14.2-q14.3, which encodes *DLEU1* (Deleted In Lymphocytic Leukemia 1), *DLEU2* (Deleted In Lymphocytic Leukemia 2), miR-15 and miR-16 [46], during distinct stages of OC maturation, including fusion and bone resorption. Currently, the role of these closely located ncRNAs in non-tumorigenic-derived bone diseases and/or cells, specifically in OCs, has not yet been disclosed. Here, we performed an in-depth analysis of the OC phenotypes regulated by *DLEU1* and miR-16 in a stage-specific manner. Taken together, our findings reveal an antagonistic relationship between *DLEU1* and miR-16 when considering OC fusion. We also provide comprehensive insights into the impact of these molecules on individual fusion events. Moreover, our study highlights the distinct effects of *DLEU1* and miR-16 on bone resorption, with *DLEU1* primarily influencing the resorption speed during pit formation, while miR-16 was shown to affect both resorption modalities.

## Results

### *DLEU1* and miR-16-5p expression levels are dysregulated during osteoclastogenic differentiation and correlate with osteoclastogenic markers

**Fig. 1:**
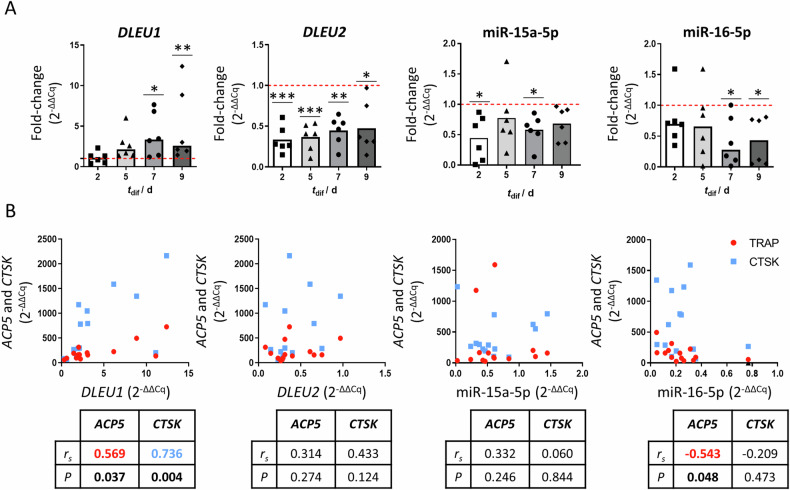
*DLEU1*, *DLEU2*, miR-15a-5p, and miR-16-5p expression levels during osteoclastogenic differentiation of human primary OCs. Human primary monocytes, isolated from buffy coats (males ≥50 years old) using the BD Imag Anti-Human CD14 Magnetic Particles kit were cultured for 2 days with 25 ng/mL of M-CSF (t_dif_: day 2) and 7 additional days with 25 ng/mL of M-CSF and 25 ng/mL of RANKL (t_dif_: day 5, 7 and 9). Expression levels are shown as a fold-change of day 0 levels (isolation day). **A** *DLEU1*, *DLEU2*, miR-15a-5p, and miR-16-5p expression levels during osteoclastogenic differentiation (*N* = 6). **B** Correlation of the expression levels of *DLEU1*, *DLEU2*, miR-15a-5p, and miR-16-5p with osteoclastogenic markers (*ACP5* and *CTSK*) (*N* = 14). Each dot represents the expression levels obtained from OCs generated in vitro from each individual donor. For each scatterplot, the Spearman correlation coefficient and significance levels are shown (red: *ACP5* and blue: *CTSK*).

To identify new potential therapeutic targets for bone diseases caused by increased resorption activity, such as osteoporosis, the expression of ncRNAs located at chr13q14.2, specifically *DLEU1*/*DLEU2*/miR-15a/miR-16-1 (Supplementary Fig. 1), was evaluated during the 9 days of osteoclastogenic differentiation from six human blood donors. *DLEU1* was significantly overexpressed at days 7 and 9, while *DLEU2* was downregulated since day 2 (Fig. 1A). To validate these results, monocytes isolated from eight additional independent donors were cultured using a different protocol with extended periods of osteoclastogenesis. The results confirmed that *DLEU1* is significantly upregulated during differentiation but could not confirm the observed downregulation of *DLEU2* (compare Fig. 1A with Supplementary Fig. 2). Importantly, *DLEU1*, but not *DLEU2* was positively correlated with the osteoclastogenic markers *ACP5* (TRAcP5 encoding gene) (*r*_*s*_ = 0.569; *p* = 0.037) and *CTSK* (*r*_*s*_ = 0.736; *p* = 0.004) (Fig. 1B) on day 9 of differentiation. The miRNAs were downregulated: miR-15a was significantly downregulated at days 2 and 7 of differentiation, whereas miR-16 was consistently decreased at days 7 and 9 (Fig. 1A). Notably, the expression of miR-16, but not miR-15a, negatively correlated with *ACP5* expression (*r*_*s*_ = −0.543; *p* = 0.048), suggesting a negative regulation of osteoclastogenic differentiation (Fig. 1B). Considering these results, *DLEU1* and miR-16 were selected for further assessment of their impact on the multinucleation, cell fusion and bone resorption processes of OCs.

### Silencing of *DLEU1* and overexpression of miR-16 impair OCs’ fusion through distinct mechanisms

**Fig. 2:**
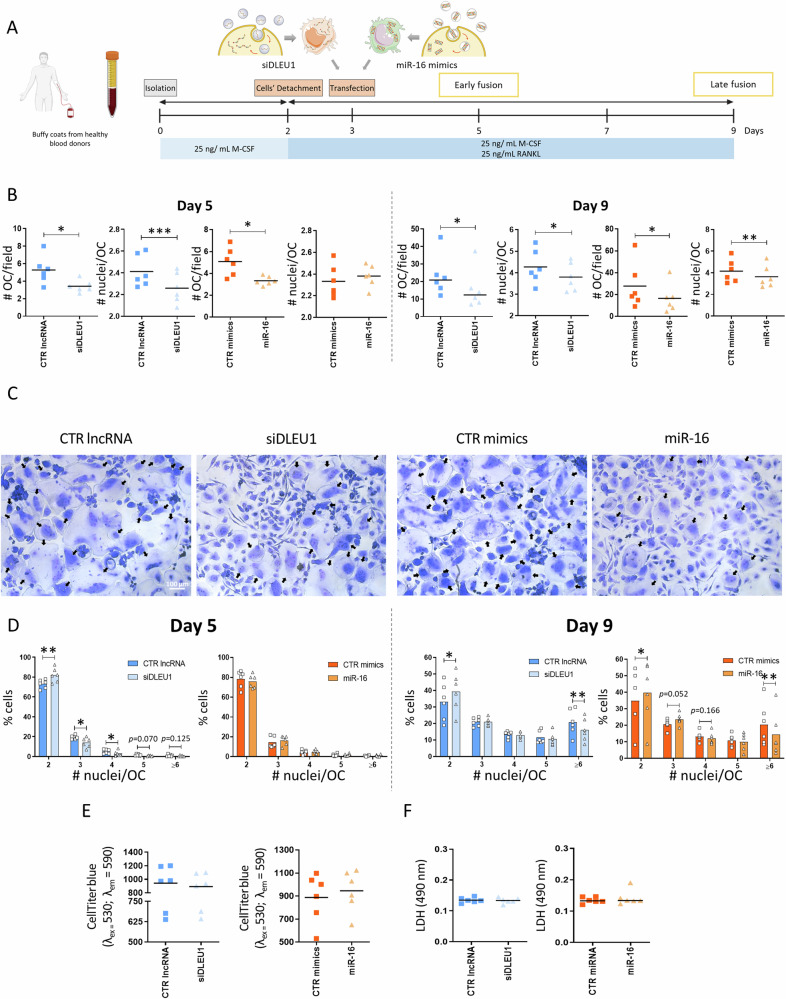
Impact of *DLEU1* and miR-16 on the multinuclearity of human primary OCs. **A** Experimental setup was used to assess the impact on the fusion capacity and multinucleation; siDLEU1-OCs: OCs transfected with a siRNA against *DLEU1* and miR-16-OCs: OCs transfected with miR-16 mimics. **B** Impact on the number of OCs per field (# OC / field) and number of nuclei per OC (# nuclei/OC) (*N* = 6), at days 5 and 9 of the differentiation process. Each dot represents the mean of a minimum of 8 wells per condition (7 fields per well) and donor. **C** Representative images of OCs transfected with CTR lncRNA, siDLEU1, CTR mimics or miR-16 mimics at day 3 and left to differentiate until day 9. Black arrows highlight the multinucleated OCs. **D** Histogram with the distribution of the percentage of the number of OCs with a specific number of nuclei at early and late fusion stages (*N* = 6). Each dot represents the mean of a minimum of 8 wells per condition (7 fields per well) and donor. **E** The metabolic activity of OCs was determined through the CellTiter-blue assay (*N* = 6). **F** Determination of the LDH levels in the conditioned media at day 5, 2 days after the transfection (*N* = 6).

To investigate the potential involvement of *DLEU1* and miR-16 in OC fusion, human precursors of OCs (pre-OCs) were transfected with either a small interfering RNA (siRNA) against *DLEU1* (siDLEU1-OC) or a miR-16 mimics (miR-16-OC) at day 3 of the differentiation process (Fig. 2A). The results revealed significant downregulation of *DLEU1* and an increase of miR-16, confirming a successful transfection (Supplementary Fig. 3). Cell fractionation showed that the *DLEU1* transcript, contrary to that of other typical nuclear lncRNAs, such as *XIST* and *MALAT1*, is located mainly in the cytoplasm (Supplementary Fig. 4).

Silencing of *DLEU1* and overexpression of miR-16 led to an impairment in the number of OCs formed per field (# OC/field), both at days 5 and 9 of differentiation (Fig. 2B, C). Accordingly, this inhibition was consistently observed within the experimental replicates of each donor (Supplementary Fig. 5). When the number of nuclei per OC (# nuclei/OC) was analyzed, the results showed a significant decrease for siDLEU1-OCs at days 5 and 9. Regarding miR-16-OCs, the # nuclei/OC was not impacted at early stages (day 5), with differences being noticed only at later stages (day 9), indicating a stage-specific regulation (Fig. 2B). A detailed analysis of the abundance of OCs with a specific number of nuclei revealed a predominance of bi-nucleated OCs, regardless of the condition, compared with OCs with more than 3 nuclei, at day 5, as expected for early stages of osteoclastogenesis (Fig. 2D). Inhibition of *DLEU1* resulted in an enrichment in bi-nucleated OCs in detriment of more mature OCs (with 3 and 4 nuclei) (day 5, Fig. 2D), which is in line with the decrease observed in the # nuclei/OC at the same time-point (day 5, Fig. 2B). In contrast, no statistically significant differences were observed after transfection of pre-OCs with miR-16 mimics compared to the control condition (at day 5, Fig. 2D). Consistent with the findings observed at day 9 (Fig. 2B) regarding the # nuclei/OC, results showed that both downregulation of *DLEU1* and overexpression of miR-16 mediated cell fusion primarily through an increase of OCs containing 2 nuclei, in detriment of a marked decrease of more matured OCs (with six or more nuclei) (Fig. 2D).

To test a potential synergistic effect on multinuclearity between the downregulation of *DLEU1* and increased levels of miR-16, pre-OCs were co-transfected with both siDLEU1 and miR-16 mimics. Using pre-OCs from the same donor, a robust decrease of the # nuclei/OC and of the # OC/field were observed for both end-points (days 5 and 9; Supplementary Fig. 6A, B). This solid decline was consistently observed in cells from three different donors. Co-transfection with siDLEU1 and miR-16 mimics resulted in a mixture of phenotypes, rather than a synergistic effect obtained when pre-OCs were transfected separately with either siDLEU1 or miR-16 mimics (compare Supplementary Fig. 6A with Fig. 2B). Moreover, the results showed that metabolic activity was not affected by siDLEU1 or miR-16 mimics (Fig. 2E). Additionally, no differences in the LDH levels released into the conditioned media were found between the siDLEU1, miR-16 mimics and control groups, excluding a cytotoxic effect (Fig. 2F).

### Proteome profiling unveils that *DLEU1* and miR-16 affect OC fusion through independent mechanisms at early time-points

**Fig. 3:**
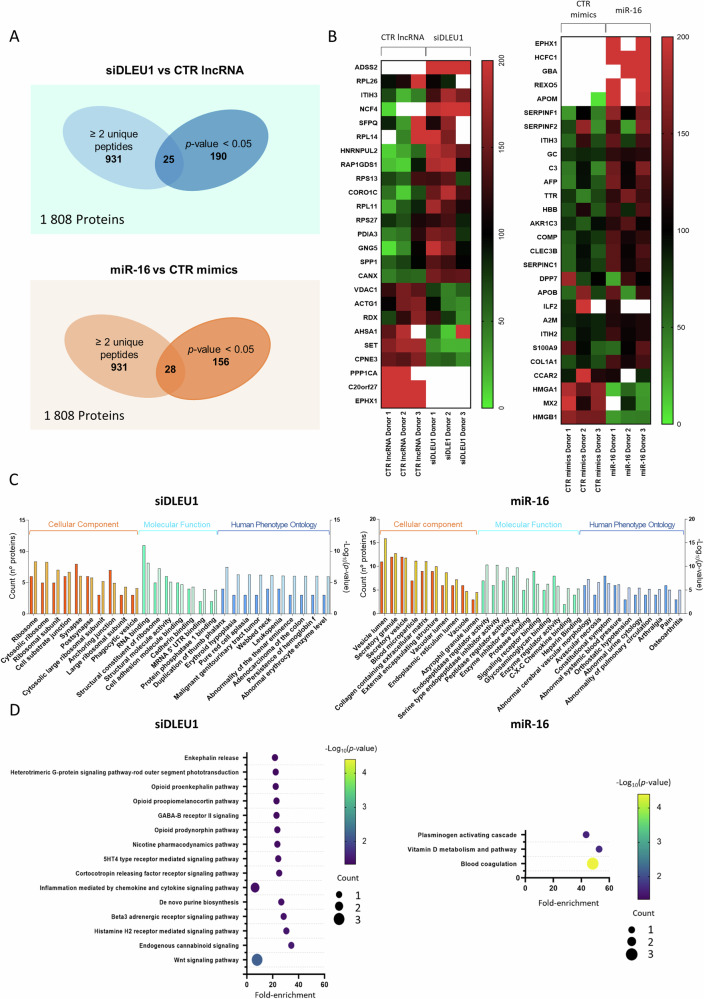
Proteomic profiling of *DLEU1* and miR-16 downstream targets and processes. **A** Venn diagrams showing the proteins that are differentially expressed after transfection with siDLEU1 and miR-16 mimics. **B** Heatmaps showing the differentially expressed proteins at day 5, with ≥2 unique peptides from three different donors after silencing *DLEU1* and overexpression of miR-16. **C** GSEA software (Gene Set Enrichment Analysis) of the differentially expressed proteins. The *x*-axis shows the number of proteins and the *y*-axis the −Log_10_(*p*-value). **D** Pathways predicted to be enriched when considering the differentially expressed proteins using PANTHER.

To screen for specific proteins regulated by *DLEU1* and miR-16, a mass spectrometry-based quantitative proteomic analysis was performed in siDLEU1- and miR-16-OCs at early stages of osteoclastogenesis (day 5, see Fig. 2A). When considering a minimum requirement of two unique peptides per detected protein, a total of 931 proteins were identified (Fig. 3A). Results show that, when compared with the respective controls, 25 proteins (16 upregulated and 9 downregulated) were differentially expressed in the siDLEU1-OCs (*p* < 0.05, Fig. 3A, B), whereas 28 (25 upregulated and 3 downregulated) were significantly impacted in miR-16-OCs (*p* < 0.05, Fig. 3A, B). Among the differentially expressed proteins, only 2 were simultaneously impacted by the silencing of *DLEU1* and miR-16 mimics (Supplementary Fig. 7), namely, Epoxide Hydrolase 1 (EPHX1; P07099) and Inter-alpha-trypsin Inhibitor Heavy chain H3 (ITIH3; Q06033). EPHX1 exhibited the opposite tendency between conditions, while ITIH3 was upregulated in both conditions, suggesting that *DLEU1* and miR-16 act on OC differentiation through independent mechanisms. To validate our proteomic results, the levels of several differentially expressed proteins were assessed through ICC. These included both upregulated and downregulated proteins for each condition. Consistent with the proteomic results, a decrease in VDAC1 (siDLEU1-OCs) and HMGA1 (miR-16-OCs) was observed, while AFP levels were consistently increased (miR-16-OCs) across the three independent donors tested (Supplementary Fig. 8). Additionally, SPP1 protein levels were increased in 2 out of the 3 donors (Supplementary Fig. 8).

An enrichment analysis was performed using only the differentially expressed proteins to evaluate the impacted processes and functions. For siDLEU1-OCs, the differentially expressed proteins were associated with ribosomal complexes (such as “Ribosome”, “Cytosolic ribosome”, “Ribosomal subunit” and “Large ribosomal subunit”), and “Phagocytic vesicle” (Cellular Components); “Cell adhesion molecule binding” and “Cadherin binding” (Molecular Function); and numerous blood malignancies (Human Phenotype Ontology) (Fig. 3C). On the other hand, when considering miR-16-OCs, the differentially expressed proteins were related to secretory activity, such as “Secretory granule”; “Secretory vesicle”; “External encapsulating structure” and “Vacuole” (Cellular Component); “Signaling receptor binding”, “Protease binding”, “Glycosaminoglycan binding” and “C-X-C Chemokine binding” (Molecular Function); and osteoarticular diseases, including Arthralgia and Osteoarthritis (Human Phenotype Ontology) (Fig. 3C).

Interestingly, 7.9% of these proteins were associated with the “Wnt signaling pathway” (P00057; siDLEU1; Fig. 3D) and 12.5% with the “Vitamin D metabolism and pathway” (P04396; miR-16; Fig. 3D). Notably, proteomic analysis also revealed that depletion of *DLEU1* led to overexpression of the Neutrophil Cytosolic Factor 4 (NFC4) protein, which is linked to the “*osteoclast differentiation”* pathway according to the Kyoto Encyclopedia of Genes and Genomes—KEGG (I04380; Supplementary Fig. 9), and an impairment of the Actin Gamma 1 (ACTG1) protein, which is one of the proteins responsible for phagocytosis and formation of the phagocytic cup in the “*phagosome*” pathway (I04145; Supplementary Fig. 9).

### Time-lapse recordings detail the impact of *DLEU1* and miR-16 on the OC fusion type and fusion pairs

**Fig. 4:**
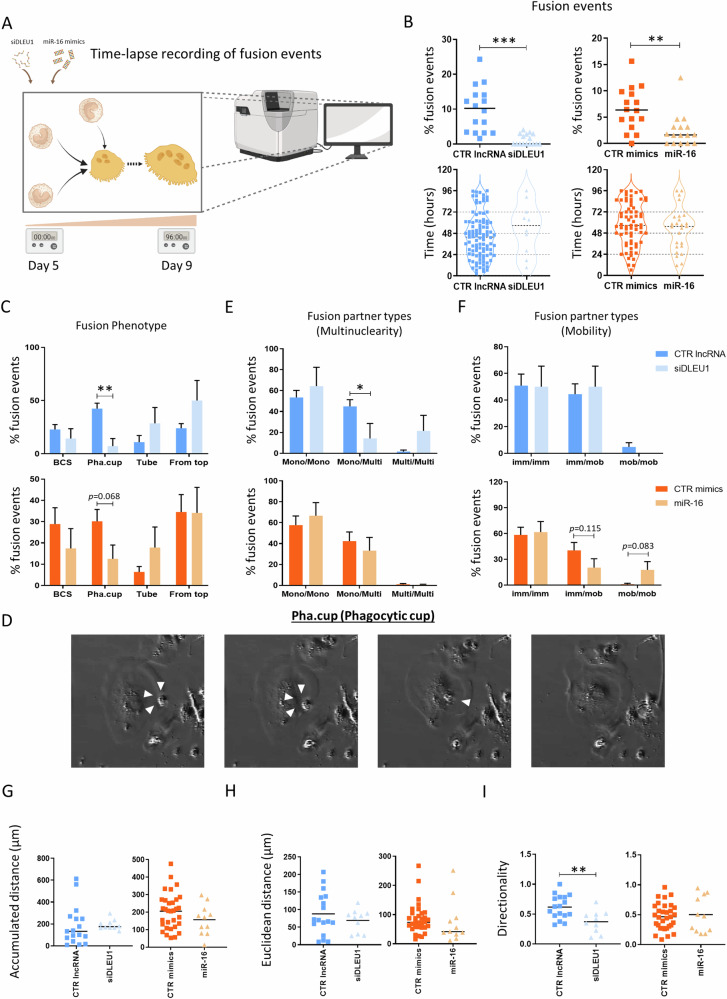
Time-lapse analysis of the cells undergoing fusion and characterization of the corresponding fusion events. **A** Schematic illustration of the time-lapse recordings performed to assess the impact on cell fusion. **B** Percentage of OCs undergoing fusion and the respective time point at which these fusion events occur. **C** Analysis of the frequency of fusion events with respect to fusion modality and **D** representative images of the phagocytic cup (*Pha.cup*) fusion modality. **E** Analysis of the frequency of fusion partners classified according to their nuclearity (mononuclear cells with mononuclear cells: mono-mono; mononuclear cells with multinucleated cells: mono-multi; multinucleated cells with multinucleated cells: multi-multi). **F** Analysis of the frequency of the fusion partners classified according to their mobility. **G** Accumulated distance; **H** Euclidean distance and **I** Directionality of the mobile OCs undergoing fusion. The graphs provided depict data from 16 different fields from a single donor, representative of a total of four donors.

Time-lapse analysis was used to track and follow the behavior of OCs at the single cell level, throughout 24 h, over the course of 4 days using cells from four different donors (Fig. 4A). In line with the results obtained with May-Grünwald staining (Fig. 2C, D), a significant decrease in fusion events was observed in siDLEU1- and miR-16-OCs (Fig. 4B). Nonetheless, no differences were detected regarding the time at which the events occurred (Fig. 4B). The fusion modalities of the OCs were also investigated (Fig. 4C, D and Supplementary Fig. 10). Regarding the fusion phenotype, the results showed a consistent decrease in the *phagocytic cup* mode (*Pha.Cup*) for siDLEU1-OCs, compared to the control condition, for the four donors used (Fig. 4C, D), while an increase in the *from top* fusion mode was observed in three out of the four donors (Fig. 4C and Supplementary Fig. 10). However, no differences were found in the fusion phenotype when comparing miR-16-OCs with the control condition. Concerning the multinuclearity of the fusion partners, siDLEU1-OCs exhibit a specific impairment of fusion between mono and multinucleated OC, whilst there were no differences for miR-16-OCs (Fig. 4E and Supplementary Fig. 11).

Since the fusion capacity of the OCs can be compromised if cell mobility is affected, this parameter was also investigated for the OCs that fuse. No significant differences were found for either siDLEU1- or miR-16-OCs. However, there was a tendency for a reduction in the fusion between immobile and mobile fusion partners (imm/mob) and an increase in the frequency of fusion between mobile and mobile pairs (mob/mob) for miR-16-OCs (Fig. 4F). Furthermore, the accumulated traveled distance (Fig. 4G), the euclidean distance (Fig. 4H), and the directionality (Fig. 4I) were measured in OCs at early stages of differentiation (day 5). Overall, no differences were observed for any of these parameters (Fig. 4G, H), except for the directionality in the siDLEU1-OCs, which traveled less linearly (Fig. 4I). Overall, these results suggest that siDLEU1, but not miR-16, has an impact on OC fusion types, fusion partners and directionality of movement.

### Effect of *DLEU1* and miR-16 on the migratory capacity of OCs

**Fig. 5:**
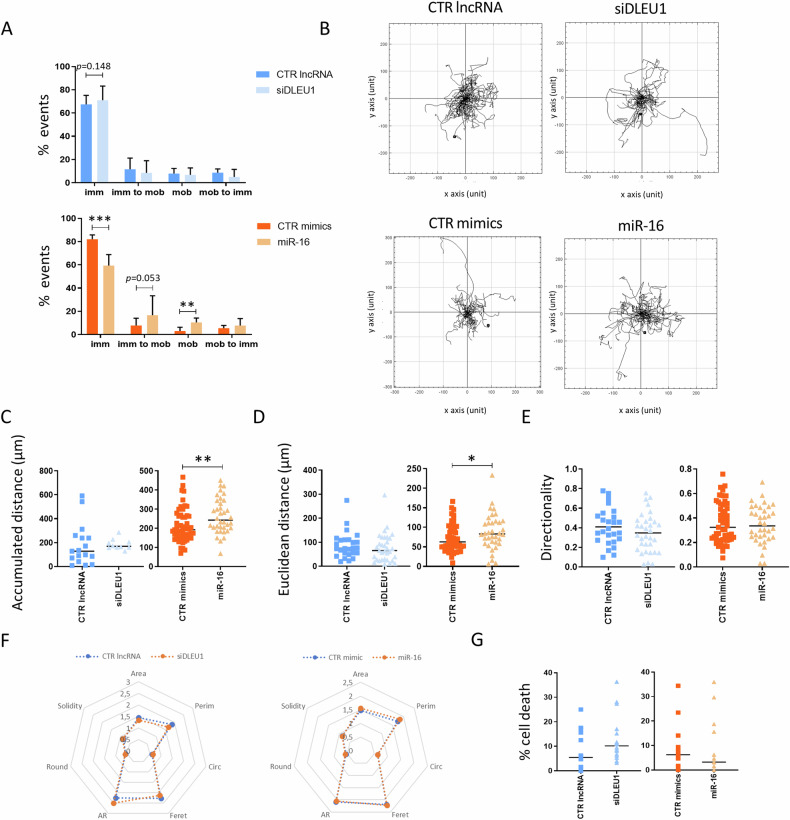
Time-lapse analysis of the migratory capacity, cell death, and division of non-fusing and fusing cells. **A** Analysis of the frequency of cells classified according to a specific migration pattern. **B** Representative tracks of mobile OCs per condition. **C** Accumulated distance; **D** Euclidean distance and **E** Directionality of the mobile OCs. **F** Cell area/shape parameters and; **G** Percentage of cell death. The graphs provided depict data from 16 different vision fields from a single donor, representative of a total of four donors.

A comprehensive analysis encompassing all OCs (fusing and non-fusing) was then carried out, instead of solely focusing on OCs undergoing fusion, as performed in the previous section. Interestingly, results showed a lower percentage of immobile cells and an increase of OCs in the mobile regime in the miR-16-OCs group, when compared with CTR mimics (5 A and 5B). No differences were found for siDLEU1 (Fig. 5A, B). Additionally, increases in the accumulated distance (Fig. 5C) and in the Euclidean distance (Fig. 5D) were observed for miR-16-OCs, while no differences were observed for siDLEU1-OCs (Fig. 5C–E). A more general approach demonstrated that the shape parameters of the OCs were not altered by modulation of *DLEU1* or miR-16 levels (Fig. 5F). Moreover, in agreement with the LDH measurements, no differences were detected regarding the number of cells dying (Fig. 5G). Taken together, the results suggest that miR-16 increases OC mobility, leading to enhanced migration (both accumulated and Euclidean distance), compared with the control group (Fig. 5A, C, and D). These findings may indicate that since miR-16-OCs fuse less, the cells need to search more for the respective fusion partner.

### Differential impact of *DLEU1* and miR-16 on resorption modes

**Fig. 6:**
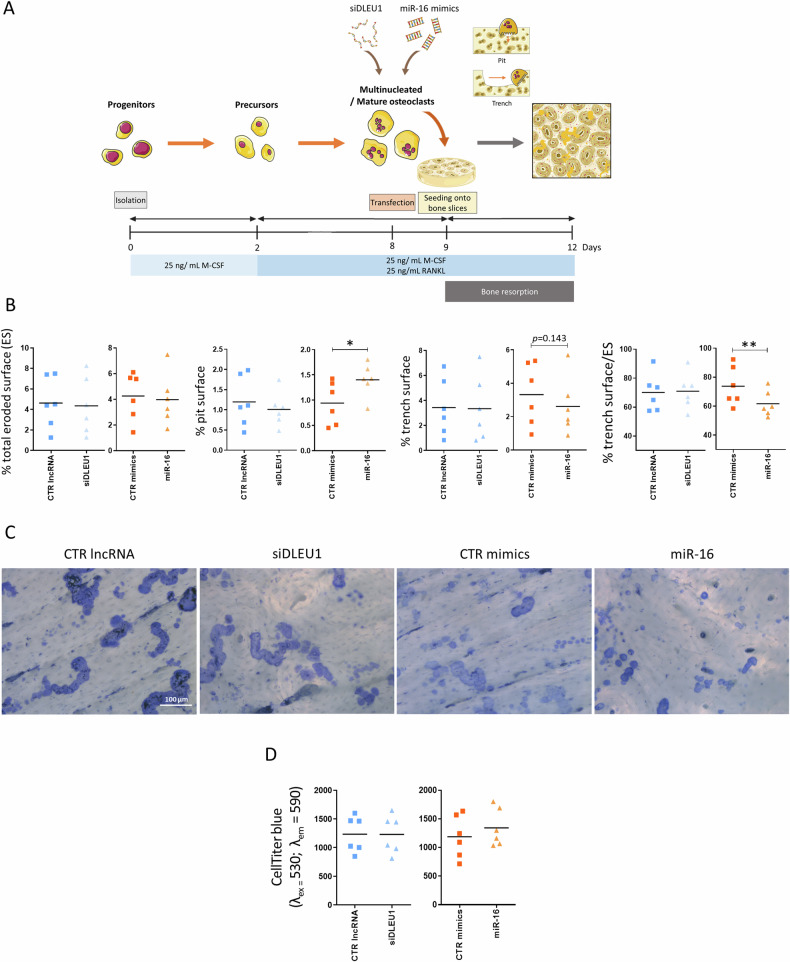
Impact of *DLEU1* and miR-16 on bone resorption. **A** Scheme of the experimental design was used to assess the impact on bone resorption. **B** Determination of the eroded surface generated by OCs after *DLEU1* silencing and miR-16 overexpression (*N* = 6) (5 slices per condition and per donor). **C** Representative images of the resorbed area formed by OCs transfected with CTR lncRNA, siDLEU1, CTR mimics, or miR-16 mimics at day 8, seeded onto bone slices at day 9 and left to resorb until day 12 (5 slices per condition and per donor). **D** The metabolic activity of OCs as determined by CellTiter-blue assay (*N* = 6) after 3 days of culture on bone slices.

We next investigated whether *DLEU1* and/or miR-16 affect bone resorption. The expression profiles of *DLEU1* and miR-16 were evaluated 72 h after seeding matured OCs onto bone slices and allowing them to resorb bone (Supplementary video). The results revealed a significant reduction in the miR-16 expression levels for all six donors, while the expression of *DLEU1* exhibited heterogeneity and non-statistically significant differences, but five out of the six donors presented an upregulation (Supplementary Fig. 12). These findings suggest that miR-16 mimics, rather than siDLEU1, have a greater impact on bone resorption capacity. To further investigate this phenomenon, mature OCs were transfected with siDLEU1, miR-16 mimics, or the respective controls, on day 8 of the differentiation process. The transfected OCs were then seeded on top of bone slices on the next day (day 9) and allowed to resorb bone until day 12 (Fig. 6A).

End-point analyses of the bone slices on day 12 showed that neither the silencing of *DLEU1* nor the overexpression of miR-16 affected the percentage of the total eroded surface (Fig. 6B, C). For siDLEU1-OCs, no differences were detected regarding the resorption modalities (pits and trenches) (Fig. 6B). In contrast, miR-16-OCs exhibited a shift in the resorption mode toward an increase of OCs forming pits, in detriment of those forming trenches, which were significantly decreased (Fig. 6B). Finally, metabolic activity was assessed at day 12 and neither siDLEU1-OCs nor miR-16-OCs were affected (Fig. 6D).

### Time-lapse recordings detail the impact of *DLEU1* and miR-16 on each resorption mode

**Fig. 7:**
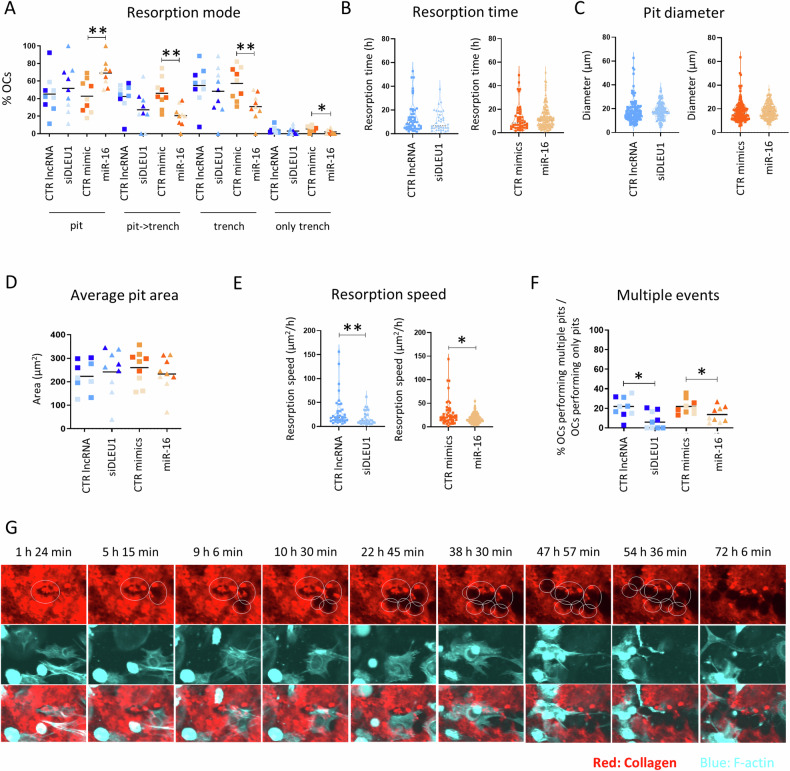
Time-lapse analysis of the resorption mode and resorptive capacity of OCs performing pits. **A** Percentage of OCs categorized according to the resorption mode. Each dot represents the mean obtained from each video analyzed (3 per donor). For each condition, dots with the same shading correspond to videos from the same donor. (OCs that only form pits: pit; OCs that form trenches that started as pit: pit->trench; OCs that form trenches without starting with a pit: only trench; OCs that form trenches, either starting with a pit or not: trench). **B** Resorption time is taken by the OCs to make a pit. The resorption time of each OC from the 3 recordings analyzed from a representative donor, doing a pit was assessed and is shown as a dot. **C** Diameter of the pit cavities. The diameter of each pit from 3 recordings was analyzed by a representative donor. **D** Average pit area. Each dot represents the mean obtained from each video analyzed (3 per donor). For each condition, dots with the same shading correspond to videos from the same donor. **E** The resorption speed of the OCs making pits. The results are based on analyses of 3 recordings from a representative donor. **F** Percentage of OCs that perform multiple pits when considering only the pit-forming OCs. Each dot represents the mean obtained from each video analyzed (3 per donor). For each condition, dots with the same shading correspond to videos from the same donor. **G** Selected time-lapse images of OCs, from the CTR lncRNA condition, making multiple pits over the course of 72 h.

To further elucidate the impact of these ncRNAs on parameters associated with the resorption mode of OCs and understand the variations observed in the end-point analysis, we studied the behavior of siDLEU1-OCs and miR-16-OCs over a 72 h period using cells from three independent donors. No significant differences were observed after silencing of *DLEU1* (Fig. 7A). However, an increase in the number of OCs making pits and a reduction in those making trenches, were observed for miR-16-OCs, when compared with the respective control (Fig. 7A). Regarding the OCs performing pits, no differences were observed regarding the resorption duration (Fig. 7B) and the diameter of the formed pits (Fig. 7C), for all the conditions. The average pit area also remained unaffected (Fig. 7D) by siDLEU1 or the miR-16 mimics. On the other hand, siDLEU1 and miR-16 negatively impacted OCs’ resorption speed (Fig. 7E) and the ability of a single OC to perform multiple pits (Fig. 7E–G).

**Fig. 8:**
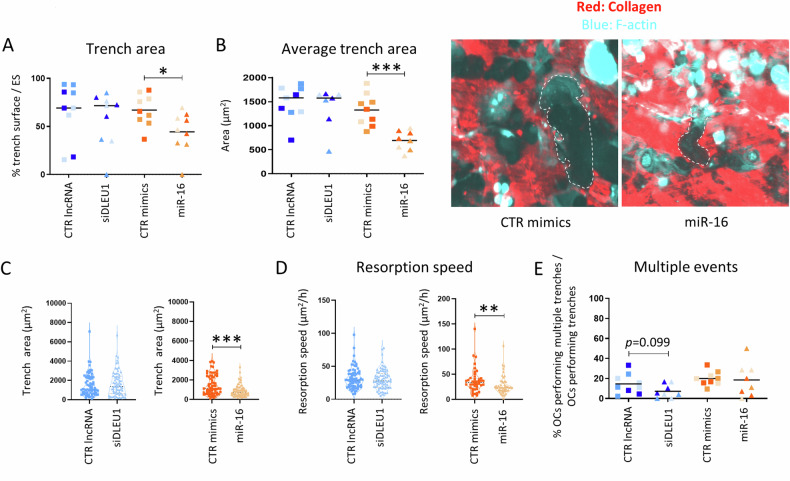
Time-lapse analysis of the resorptive capacity of OCs performing trenches. **A** Percentage of the area covered by trenches normalized against an eroded surface (ES). Each dot represents the mean obtained from each video analyzed (3 per donor). For each condition, dots with the same shading correspond to videos from the same donor. **B** Average area of the trenches (left) and representative time-lapse images (right). For each condition, dots with the same shading correspond to data obtained from videos with cells from the same donor. **C** The area of individual trenches based on the analyses of 3 recordings from a representative donor. **D** The resorption speed of OCs making individual trenches was obtained from analyses of 3 recordings from a representative donor. **E** Percentage of OCs that perform multiple trenches when considering only trench-forming OCs. Each dot represents the mean obtained from each video analyzed (3 per donor). For each condition, dots with the same shading correspond to videos from the same donor.

Finally, a series of analyses focusing on the impact of both ncRNAs on trench mode was conducted. Time-lapse results showed no differences for siDLEU1-OCs, while in the miR-16-OCs group, a significant reduction in the area resorbed was observed for OCs making trenches (Fig. 8A), corroborating the results obtained in the end-point analysis (Fig. 6B). This reduction may be partly explained by the decrease in the average area resorbed per trench and by the negative impact on the resorption speed in the miR-16-OCs group, compared with the control group (Fig. 8B–D). Despite being slower, miR-16-OCs still maintained the ability to perform multiple trenches during the time-lapse recordings (Fig. 8E). Regardless of the parameters studied, no significant changes were detected after manipulating the levels of *DLEU1* (Fig. 8A–E). Overall, these results indicate that the impact of siDLEU1 on bone resorption is mainly observed on OCs making pits, while miR-16 affects both pit and trench formation.

## Discussion

Over the past two decades, the chromosomal region 13q14, housing the *DLEU1* and the *DLEU2*/miR-15a/16-1 cluster, originally recognized as tumor suppressors [47–50], has emerged as a focal point of considerable attention in the context of several diseases. Notably, the discovery of deletions of miR-15 and miR-16 in chronic lymphocytic leukemia (CLL) [51] marked a pivotal milestone that ignited subsequent urges in research within the ncRNA field. In bone biology, modulation of ncRNAs has been shown to promote bone regeneration [2], but the role of ncRNAs in OC physiology and bone resorption remains far less explored. Presently, there are more than a dozen ongoing clinical trials exploring the therapeutic potential of miRNAs and siRNAs [52–55]. In contrast, the number of clinical trials involving lncRNAs remains limited, primarily due to a lack of comprehensive understanding of their functions [56]. In this study, we conducted a comparative analysis of the molecular functions of the lncRNA *DLEU1* and the small ncRNA miR-16-5p in OC biology. For this purpose, we exclusively used human primary monocyte-derived OCs, deliberately avoiding the use of cell lines. This strategy effectively accounts for the natural variations between different blood donors [16, 18, 57], thus providing a closer ex-vivo representation of the human in vivo settings. In this study, monocytes isolated from male donors were used because OCs derived from males tend to exhibit a higher degree of aggressiveness, when compared to those derived from females [18, 20, 58] since we hypothesized that our treatments would hinder OC fusion and/or OC bone resorption capacity. Additionally, monocytes were obtained from older individuals since there is a notable increase in OC activity and higher levels of active CTSK in this demographic group [7, 59]. Despite the absence of a complex in vivo microenvironment, the behavior of OCs in vitro has been shown to match in vivo characteristics, such as age, menopausal status, level of type I procollagen N-terminal propeptide (PINP), and C-terminal telopeptide of type I collagen (CTX), as well as the lifestyle of the donors (e.g., smoking habits) [7, 16, 18, 60, 61].

To determine the functional processes affected by the modulation of *DLEU1* and miR-16, we not only performed end-point analyses but also addressed the impact of modulating *DLEU1* and miR-16 on OCs at the single-cell level by conducting time-lapse analyses. This approach enabled us to closely examine individual fusion events. Considering that multinucleation is not a random process and can be achieved through different fusion routes [30, 32, 62, 63], this approach was fundamental for answering the question of how a certain end-point result was attained. It also allowed us to determine the involvement of fusion-related factors, which would otherwise be challenging to quantify. In our analysis, we also included OCs with two nuclei to avoid missing the first fusion event. The absence of differences in LDH levels and metabolic activity ruled out an effect on OC fusion due to an impact on cell viability.

The recordings revealed an impact on fusion modalities, particularly a reduction in the *Pha.cup* fusion mode in siDLEU1-OCs. This impairment may be partially attributed to the impact of siDLEU1 on the levels of the cytoskeletal protein ACTG1, which is linked to the term Phagosome (I04145), which includes the endocytic/phagocytic pathways, that are reported to be implicated in the fusion of OCs and macrophages [64, 65] through mechanisms matching the *Pha.cup* mode. Interestingly, the impairment of endocytosis observed after *Actg1* knockout [66] also strengthens this hypothesis. Additionally, a decrease was observed in the fusion events between mono- and multinucleated partners when *DLEU1* levels were decreased. This reduction is consistent with the decreased number of OC with ≥3 nuclei, observed at both early and late stages of osteoclastogenesis. Together with the higher number of bi-nucleated OCs in the siDLEU1 condition, these results suggest an impairment of the fusion capacity through an obstruction in the fusion between smaller OCs. When compared to miR-16-OC, no differences in the multinucleation were found at early fusion stages, nevertheless, an accumulation of bi-nucleated OC and a decrease in OC with more than 6 nuclei was observed at late fusion stages. Additionally, no differences were observed regarding the fusion partners and modes for the miR-16-OC group. These results collectively suggest that miR-16 specifically targets pre-OCs, reducing their ability to fuse. Interestingly, despite the reduction in fusion events for miR-16-OC, the data revealed an increase in cell migration. While this may initially appear contradictory, as increased migration typically implies a greater potential for fusion, the higher migratory profile may indicate that cells need to search more extensively for their respective fusion partners. In a study on giant cell tumors, Sang et al. reported that miR-16 promoted OC formation in mice bone marrow-derived macrophages [67]. Another miRNA from the same family, miR-195 [68], has also been reported to negatively regulate the expression of key osteoclastogenic genes [69]. These reports further support our findings, demonstrating that miR-16 impairs OC formation.

To support the potential of ncRNA as a new class of anti-resorption molecules, we conducted an in-depth investigation into the impact of miR-16 mimics and siDLEU1 on bone resorption, a unique feature of the OCs. Although these molecules do not impact the total area eroded by the OCs, they have a differential impact on the resorption mode, which has been shown to be clinically relevant and associated with the severity of OC bone resorption [7, 16]. Specifically, Vanderoost et al. reported that for matching levels of erosion, the bone is more fragile when trenches are predominant [70]. Accordingly, OCs making trenches have higher erosion rates, create deeper cavities and have increased levels of active CTSK, reflecting a more aggressive form of bone resorption than those making pits [20, 71]. Additionally, the importance of distinguishing resorption cavities between pits and trenches is even more evident when considering that the proportion of surface covered by trenches is increased by glucocorticoids [72] and inhibited by odanacatib [20], the latter being well known to elevate the bone mineral density in postmenopausal women with reduced bone mass [73]. In our findings, only miR-16-OCs, not siDLEU1-OCs, exhibited a negative impact on the trench resorption mode, specifically concerning the average trench area and resorption speed. Regarding pit formation, while both siDLEU1 and miR-16 mimics influenced this resorption modality, the differences were more prominent for miR-16-resorbing-OC, culminating in an increase in the total eroded area covered by pits. Thus, delivery of miR-16 mimics into matured OCs seems a more promising approach to decrease the OCs’ aggressiveness, though not abolishing it.

Mass spectrometry analysis provided further support for the hypothesis that siDLEU1 and miR-16 mimics while sharing some functional overlap, operate through distinct mechanisms in OCs. Interestingly, only two proteins were found to be impacted by both ncRNAs. Notably, several of the differentially expressed proteins have a role in regulating OC physiology and activity. For example, An et al. reported that mitochondrial proteins, including voltage-dependent anion-selective channel protein 1 (VDAC1), are decisive for the formation of mature OCs and play pivotal roles associated with the balance between their bone resorption activity and survival [74]. Treatment with an anti-VDAC antibody inhibited osteoclastogenesis and bone resorption in human OCs in vitro [75]. Accordingly, we observed impairment of VDAC1 in siDLEU1-OCs. On the other hand, lysosomal acid glucosylceramidase (GBA), whose inhibition was previously shown to increase the number of OCs formed [76], was identified by us as one of the top-upregulated proteins in miR-16-OC. Accordingly, Siebert et al. described miR-16 to positively regulate GBA activity and expression in human fibroblasts [77]. Furthermore, several members of the serine protease inhibitor family, specifically SERPINF1, SERPINF2, and SERPINC1, exhibited high expression levels in miR-16-OCs. However, only SERPINF1, also known as PEDF, negatively affects osteoclastogenesis, RANKL-mediated survival, and bone resorption activity [78].

Overall, both ncRNAs *DLEU1* and miR-16 hold significant promise for the development of targeted treatments to counteract bone resorption and osteoporosis-related complications, either by influencing the fusion process and/or by directly impairing the resorptive activity of the OCs.

## Materials and methods

### In vitro generation of human OCs from buffy coats

For osteoclastogenic differentiation and functional assays, CD14^+^ monocytes were purified, as previously reported, from human buffy coats (BC) of anonymous 50-65 years old male blood donors, with their informed consent, donated by the Odense University Hospital (Denmark) [7, 79, 80]. Briefly, BC was diluted, laid over Ficoll-Paque (Amersham, GE Healthcare, Little Chalfont, UK), and centrifuged at 900 × *g*, for 20 min. Peripheral blood mononuclear cells (PBMCs) were then collected and washed, and CD14^+^ monocytes were subsequently isolated by immunomagnetic separation, using the BD IMag Anti-Human CD14 Magnetic Particles—DM (BD Biosciences, San Jose, CA, USA), according to the instructions given by the supplier. Cells were seeded at a cell density of 6.7 × 10^4^ cells/cm^2^ in cell culture flasks (Greiner), in α-MEM (Thermo Fisher Scientific), supplemented with 10% (v/v) FBS (Sigma-Aldrich), 1% (v/v) Penicillin-Streptomycin (P/S, Sigma-Aldrich) and 25 ng/mL M-CSF (R&D Systems) for 2 days. Afterward, the culture media was renewed and cells were differentiated into mature osteoclasts (OCs) over the course of 7 additional days with 25 ng/mL M-CSF and 25 ng/mL RANKL (both from R&D Systems), as previously described [17, 30, 72]. An independent cohort of BC from healthy donors was collected at Hospital Universitário São João (Portugal) for validation experiments, and isolation and cell culture of these donors was performed as previously described [41].

### Oligonucleotide transfection

Human primary OCs at several stages of differentiation were transiently transfected with either a silencing RNA against Deleted In Lymphocytic Leukemia 1 (DLEU1) (siDLEU1; 25 nM; Lincode Human DLEU1 siRNA—SMARTpool; R-0200009-00-0005; Dharmacon), miR-16 mimics (miR-16; 50 nM; mirVana® miRNA mimic; MC10339; ThermoFisher Scientific), or the respective negative controls [Lincode Non-targeting Pool (CTR lncRNA; 25 nM; D-001320-10-05; Dharmacon) and mirVana™ miRNA Mimic, Negative Control #1 (CTR mimics; 50 nM; 4464058; ThermoFisher Scientific)].

The oligonucleotides were delivered using the GenMute™ siRNA transfection reagent for primary macrophages (SL100568-PMG, SignaGen Laboratories), according to the manufacturer’s protocol. Briefly, a working solution was prepared, the oligonucleotides were individually added, according to the concentrations mentioned above, and the solution was left to incubate for 15 min, to allow the transfection complexes to form, and then added to the cells.

The transfections were performed at day 3 post-monocyte isolation for fusion assays, RNA and protein collection; at day 4 post-monocyte isolation for fusion time-lapse recordings; and at day 8 post-monocyte isolation for resorption experiments (both end-point and time-lapse recordings).

### RNA isolation

Total RNA was extracted from human primary OCs using TRIzol Reagent (Invitrogen) according to the manufacturer’s instructions. The RNA concentration and purity were determined using a NanoDrop Spectrophotometer ND-1000 (ThermoFisher Scientific). Total RNA samples were stored at −80 °C until further use.

### Reverse transcription and real-time quantitative polymerase chain reaction (RT-qPCR)

For the analysis of the miRNA expression levels, TaqMan MicroRNA Reverse Transcription Kit and gene-specific stem-loop Reverse Transcription primers (Applied Biosystems) were used, according to the manufacturer’s protocol and as previously described [41, 42]. hsa-miR-15a-5p and hsa-miR-16-5p FASTA format sequence annotations were obtained from the miRbase database (http://www.mirbase.org/, Supplementary Table 1). The RT-qPCR reaction was performed under the following conditions: 10 min at 95 °C, 40 cycles of 15 s at 95 °C, and 1 min at 60 °C. The small nuclear RNA U6 was used as a reference gene.

For the analysis of the expression of coding and non-coding transcripts, RNA samples were digested with a TURBO DNA-free kit (Life Technologies), following the manufacturer’s instructions, to remove potential DNA contaminants. Buffer and Dnase were added to the samples and incubated for 30 min, at 37 °C. Then, a DNase inhibitor was added to the samples, which were subsequently vortexed and centrifuged at 10,000 × *g*, for 1 min. The supernatant containing the RNA was collected and the RNA samples were subsequently used to synthesize cDNA, using random hexamers (Life Technologies) and the SuperScript® III Reverse Transcriptase kit (Life Technologies), as already described [41, 42]. qPCR reaction was performed by adding SYBR Green PCR Mastermix (Bio-Rad), nuclease-free water, and the pair of primers corresponding to the transcript of interest (reverse and forward; Supplementary Table 2), to the cDNA, as reported [81]. The reaction was performed in a CFX Real-Time PCR Detection System (Bio-Rad), according to the following conditions: 3 min at 95 °C (denaturation) and 40 cycles of 30 s at 95 °C (cDNA denaturation), 30 s at 58 °C (annealing) and 30 s at 72 °C (extension). B-ACTIN was used as a reference gene.

Relative expression levels for each targeted miRNA and genes were calculated using the quantification cycle (Cq) method, according to the MIQE guidelines [82]. Data were analyzed using the Bio-Rad CFX Manager software and all the qPCR reactions were performed in duplicate.

### Fusion assay

At days 5 and 9 of the differentiation process, OCs were fixed with 3.7% (v/v) formalin solution and stained with Giemsa and May-Grünwald, as previously described [83]. After fixation, methanol was added to each well and left to incubate for 15 min. Cells were air dried and the May-Grünwald solution (J.T.Baker^®^), followed by the Giemsa solution (Merck), was added to the samples. Finally, the cells were washed twice with distilled water and all the multinucleated OCs (≥2 nuclei), and their respective number of nuclei, were systematically counted in 7 different random fields (assigned by a random number generator) in 8–10 replicate wells for each condition, using the Axiovert 200 microscope (Carl Zeiss).

### Bone resorption assays and quantification of bone resorption

Mature OCs (cultured for 2 days with 25 ng/mL M-CSF and for 7 additional days with 25 ng/mL M-CSF and 25 ng/mL RANKL) were detached using accutase (Biowest). Subsequently, the cells were reseeded on top of 0.4 mm thick bovine cortical bone slices (Boneslices.com) to offer a biologically relevant bone substrate, surpassing the performance of synthetic materials or dentine slices. The seeding density was 5.0 × 10^4^ cells/bone slice (5 bone slices/condition). OCs were then cultured in α-MEM supplemented with 10% (v/v) FBS, 25 ng/mL M-CSF, and 25 ng/mL RANKL for 3 additional days [79]. Before the analysis of the eroded surface and cell lysis/removal, the cellTiter-Blue® Cell viability Assay was performed. Resorption events were visualized and quantified after staining with toluidine blue, as previously described [84]. The samples were analyzed by light microscopy for the percentage of eroded surface, type of resorption pattern (pits or trenches, in accordance with published definitions [17, 72]) and number of resorption events, with a 10x objective through a 100 point grid graticule (catalog number:01A24.5075, Graticules Optics) [18, 72]. All quantifications were performed in a blinded manner with respect to the treatment.

### CellTiter-blue assay

To measure cells’ viability and activity, the CellTiter-Blue® Cell viability Assay (Promega) was used. Briefly, 10% (v/v) of the dye was added to each well and incubated for 20 min, at 37 °C, and 5% (v/v) CO_2_, protected from the light. The supernatant was then collected, transferred to a black 96-well plate (Greiner), and the fluorescence was read at an excitation wavelength of 530 nm and an emission wavelength of 590 nm, using the spectrophotometer microplate reader Synergy MX (Biotek Synergy).

### Colorimetric lactate dehydrogenase (LDH) assay

Cell viability/cytotoxicity was also evaluated through quantification of the plasma membrane damage using the CytoTox 96® Non-Radioactive Cytotoxicity Assay (Promega). The protocol was performed according to the manufacturer’s instructions. Briefly, the conversion of iodonitrotetrazolium violet (INT) into a red formazan product by LDH was measured through incubation with the substrate solution for 30 min, at RT, protected from the light. Then, a stop solution was added, and the absorbance was measured at 490 nm, using the spectrophotometer microplate reader Synergy MX (Biotek Synergy). LDH levels in the conditioned media were measured at day 5 of the differentiation process from OCs transfected at day 3.

### Protein extraction and quantification

Pre-OCs transfected with siDLEU1, miR-16 mimics, or the respective controls, at day 3 were cultured under osteoclastogenic differentiation conditions for 2 additional days. Cells were lysed in the presence of phosphatase and protease inhibitors (Thermo Scientific) at day 5 of the differentiation process, after being washed with PBS 1x. Cell lysates were clarified through a 20,000 × *g* centrifugation, for 10 min at 4 °C. The supernatants, containing the protein, were collected and protein concentration was determined using the DC Protein assay kit (Bio-Rad).

### Proteomic analysis

For the mass-spectrometry analysis, 40 μg of protein lysates from transfected OCs were used. Protein identification and label-free quantitation were performed by nanoLC-MS/MS, composed of an Ultimate 3000 liquid chromatography system coupled to a Q-Exactive Hybrid Quadrupole-Orbitrap mass spectrometer (Thermo Scientific), as previously reported [41, 81, 85]. The raw data were processed using Proteome Discoverer 2.3.0.523 software (Thermo Scientific). Protein identification was performed with the Sequest HT search engine against *Homo sapiens* entries from the UniProt database (https://www.uniprot.org/). Protein and peptide confidence levels were set to high. The analyzed samples were normalized against the total peptide signal and its quantitative evaluation was performed via pairwise comparisons of the detected peptides. Data were corrected using the Benjamin Hochberg method. Protein levels were compared using the median ratio.

A minimum of two unique peptides per identified protein were required for further evaluation and contaminants were removed. Bioinformatics and data analysis were performed with a *p* < 0.05. The differentially expressed proteins were classified according to Gene Ontology (GO) annotations and enriched pathways. The study of the terms enriched in our differentially expressed proteins was performed using Protein Analysis Through Evolutionary Relationships (PANTHER) tool [86–91]. Gene Set Enrichment Analysis (GSEA) was also used for interpreting the expression data [92, 93].

### Immunocytochemistry (ICC)

To validate some of the targets detected to be differentially expressed in the proteomic analysis, pre-OCs transfected at day 3 and left to differentiate until day 5 were washed and fixed with 40 g/L PFA for 30 min at room temperature (RT). Cells were permeabilized using 0.1% (v/v) Triton X-100 (Sigma-Aldrich) for 10 min, at RT, rinsed and blocked with 50 g/L BSA/0.1% (v/v) Triton X-100 for 1 h. Subsequently, the cells were incubated overnight at 4 °C with primary antibodies against alpha-fetoprotein (AFP, MAB1368, 1:200, R&D Sytems), high mobility group protein HMG-I/HMG-Y (HMGA1, ab252930, 1:400, Abcam), osteopontin (SPP1, 1:1000, AB1870, Merck) and voltage-dependent anion-selective channel protein 1 (VDAC1, 1:250, ab110326, Abcam). Following three 5 min washes with PBS 1×, cells were incubated with the respective secondary antibody (1:1000, anti-mouse A11020, and anti-rabbit A11008, Thermo Fisher Scientific) for 1 h at RT. The nuclei were stained using 1 μg/mL DAPI (Invitrogen) for 5 min. Negative controls included cells treated with antibody diluent alone, followed by secondary antibody incubation. The stainings were visualized and images were acquired using the Leica DMi6000 FFW microscope (Leica Microsystems). Quantifications were performed by analyzing a minimum of 4 distinct fields per condition and using the ImageJ Fiji software [94]. AFP, VDAC1, and SPP1 are represented as the area stained per cell normalized against the respective control, while HMGA1, found in the nucleus, is represented as the intensity of staining per cell normalized against the respective control.

### Time-lapse recordings and analysis of fusion assays

Three days after being reseeded into eight‐wells of a Nunc Lab‐Tek II chambered cover‐glass (Nunc−Thermo Fisher Scientific) at a density of 6.0 × 10^4^ cells/well in α-MEM, supplemented with 10% (v/v) FBS, 25 ng/mL M‐CSF, and 25 ng/mL RANKL, and one day after being transfected with either siDLEU1, miR-16 mimics or the respective controls, the OCs reached an early fusion stage (confirmed through light‐microscopy) and the time‐lapse recordings were initiated (day 5). The chambered cover‐glass was subsequently placed in the incubation chamber of the confocal Olympus Fluoview FV10i microscope (Olympus Corporation) with 5% (v/v) CO_2_ at 37 °C, for 4 days (between days 5 and 9 of the differentiation process). The media was changed 2 days after the beginning of the recordings (day 7). For each condition, four random sites were recorded (a total of 16 different sites over the course of 4 days). Time‐lapse images were acquired every 21 min for approximately 24 h, using phase contrast. This procedure was repeated for four consecutive days and for each new recording four new sites were chosen in each well.

Analysis of the time‐lapse recordings for the measurement of migration and cell shape/size, as well as to observe any fusion events, was performed using the FV10‐ASW 4.1/4.2 Viewer software (Olympus) and ImageJ [94]. When a fusion event was detected, it was characterized as previously reported [32, 62].

Data was collected from four independent donors, all with the four different experimental conditions (siDLEU1, miR-16 mimics, or the respective controls). For each donor, a total of 64 videos, reflecting a total of 1 536 h, were analyzed.

### Time-lapse recordings and analysis of resorption assays

At day 8 of the differentiation process, mature OCs were transfected with either siDLEU1, miR-16 mimics, or the respective controls. On the next day, the OCs were detached, labeled with 100 nM SiR-actin and 10 μM verapamil (both from Spirochrome), as already described [17] and reseeded onto 0.2 mm bone slices labeled with N-hydroxysuccinimide ester-activated rhodamine fluorescent dye (ThermoFisher Scientific), at a cell density of 1 × 10^5^ cells/bone slice (Boneslices.com), in 96-well plates [17, 71]. Afterward, the bone slices and the labeled transfected mature OCs were placed into eight‐wells of a Nunc Lab‐Tek II chambered cover‐glass, and bone resorption events were recorded using the confocal Olympus Fluoview FV10i microscope, at 37 °C, in 5% (v/v) CO_2_, in a humidified atmosphere. OCs were imaged using a 10x objective and a confocal aperture of 2.0. For each condition and donor, 3 random sites were recorded for 3 uninterrupted days, according to previous publications [17].

Analysis of the time‐lapse recordings for the measurement of the resorbed area and time, characterization of the resorption events and cell’s size, was performed using the FV10‐ASW 4.1/4.2 Viewer software (Olympus) and ImageJ [94]. The duration of each event was determined by calculating the number of frames elapsed from the start to the conclusion of that specific event. When resorbing in pit-mode, OCs display a round actin ring, which remains stationary during the resorption process. These resorption events were identified by the initial presence of an F-actin staining at the center of the actin ring, indicating the formation of the ruffled border, along with the start of labeled collagen removal from the bone slice surface. The termination of these resorptions events was determined by either the cessation of the ruffled border, the disappearance of the actin ring or the relocation of the OC from the event. In the case of the OC resorbing in trench mode, it is observed an initial displacement of the actin ring towards a specific side of the pit (if trench formation started after pit generation) or initiation of collagen removal that is expanding only in one direction (when the OC initiate the resorption process in trench mode right from the start). Trenches that started outside the recording window were solely used for calculating the overall resorption speeds and were not categorized based on their initiation, due to the unavailability of these data. The resorbed area of each event was determined by manually outlining the boundaries of the resulting resorption cavities, represented by the ‘black’ regions on the Rhodamine staining, using ImageJ.

Data was collected from three different donors, all with four different experimental conditions (siDLEU1, miR-16 mimics or the respective controls). For each condition and donor, a total of 216 h were analyzed (864 h when considering the four conditions).

### Statistical analysis

All graphs were performed using GraphPad Prism software, version 9 (GraphPad software). Firstly, data sets were tested, using the Shapiro-Wilk and Kolmogorov-Smirnov normality tests to determine if the data followed a parametric or non-parametric distribution. When the data passed the normality tests, a student *t*-test (2 groups) or one-way ANOVA ( > 2 groups), followed by Sidak’s multiple comparisons or Turkey’s multiple comparisons tests were used. These data are presented as means or mean ± SEM. For non-normal distribution data, non-parametric tests were used to evaluate significant differences between samples, namely two-tailed Wilcoxon matched pairs test (2 groups) or Friedman test (>2 groups), followed by uncorrected Dunn’s multiple comparison test. These data are presented as medians or median±IQR. Correlations were assessed using the Spearman’s rank (*r*_s_) correlation test. All statistical tests were two-sided. Analysis of the outliers was performed using the ROUT method (*Q* = 0.1%) and a maximum of three data points were removed. Statistical significance was achieved when *p* < 0.05 (*p < 0.05; **p < 0.01 and ***p < 0.001).

## Supplementary information

Supplementary Figures

Supplementary Tables

Supplementary Video 1

## Data Availability

Raw data is available from the corresponding author upon reasonable request.
