# Supplementary material for: Stage-specific modulation of multinucleation, fusion, and resorption by the long non-coding RNA DLEU1 and miR-16 in human primary osteoclasts
Source: Cell Death Dis. 2024 Oct 11;15(10):741. doi: 10.1038/s41419-024-06983-1 (PMC11467329; doi:10.1038/s41419-024-06983-1)
Supplement: Supplementary file 1 — Supplementary Figures [file 41419_2024_6983_MOESM1_ESM.pdf]

Supplementary files

Stage-Specific Modulation of Multinucleation, Fusion and Resorption by the Long Non-coding RNA DLEU1 and miR-16 in Human Primary Osteoclasts

Sara Reis Moura [1,2,3], Ana Beatriz Sousa [1,2,3], Jacob Bastholm Olesen [4,5,6], Mário Adolfo Barbosa [1,2], Kent Søe [4,5,6,*], Maria Inês Almeida [1,2,3,*,#]

* These authors contributed equally

Affiliations

1. i3S - Instituto de Investigação e Inovação em Saúde, Universidade do Porto, Porto, Portugal
2. INEB – Instituto de Engenharia Biomédica, Universidade do Porto, Portugal
3. ICBAS - Instituto de Ciências Biomédicas Abel Salazar, Universidade do Porto, Porto, Portugal
4. Department of Pathology, Odense University Hospital, Odense, Denmark
5. Clinical Cell Biology, Pathology Research Unit, Department of Clinical Research, University of Southern Denmark, Odense, Denmark
6. Department of Molecular Medicine, University of Southern Denmark, Odense, Denmark

#Corresponding author

Address: Rua Alfredo Allen, 208, 4200-135 Porto, Portugal

Email: ines.almeida@i3s.up.pt

Phone: +351 220 408 800

This file contains 12 Supplementary Figures.

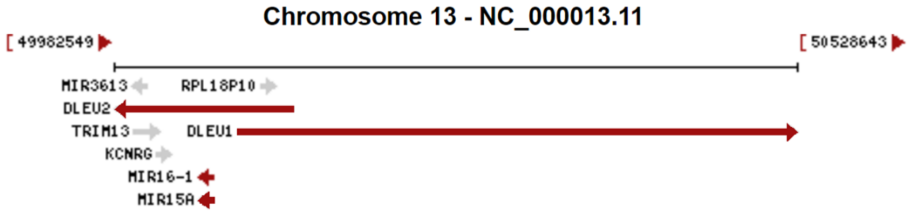

Supplementary Figure 1. Schematic representation of the *DLEU1*/*DLEU2*/*miR-16-1*/*miR-15a* locus.

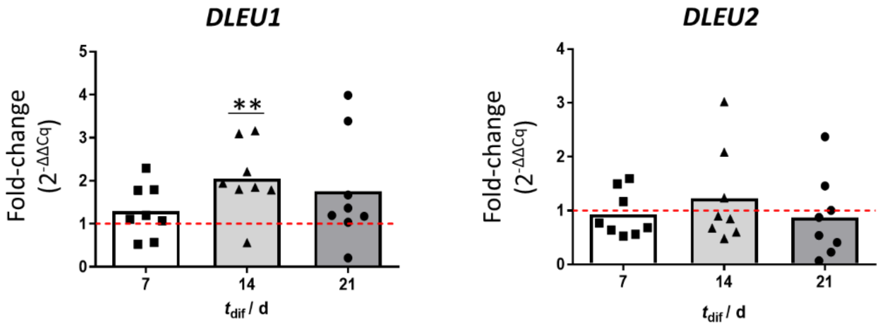

Supplementary Figure 2. *DLEU1* and *DLEU2* expression levels during osteoclastogenic differentiation of human primary monocytes isolated using the RosetteSep™ Human Monocyte Enrichment Cocktail (*N*=8), in comparison to day 0 (isolation day).

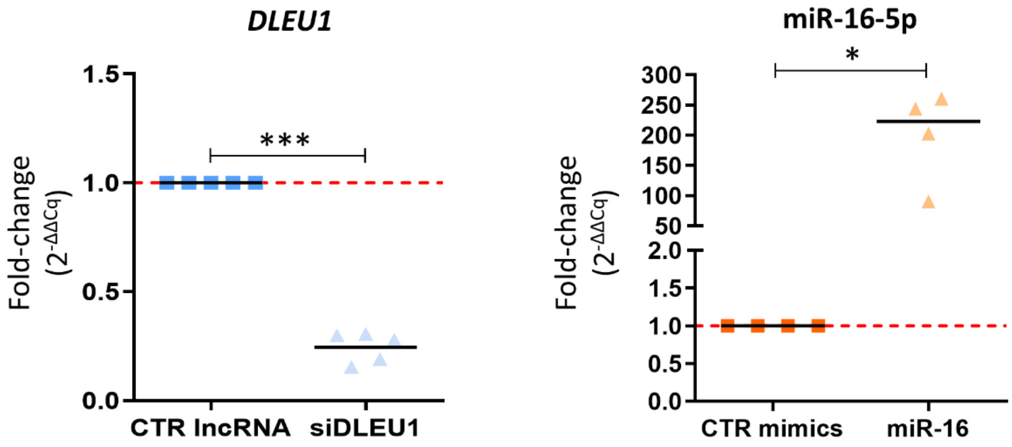

Supplementary Figure 3. *DLEU1* and miR-16 transcript levels ($2^{-\Delta\Delta Cq}$) on day 5, in OCs after transfection at day 3 with siDLEU1 (*N*=5), miR-16 mimics (*N*=4), or the respective controls. Each dot represents a different donor.

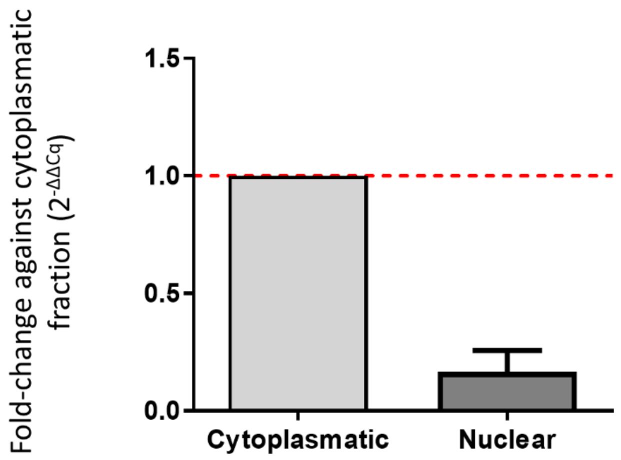

Supplementary Figure 4. *DLEU1* subcellular location in monocytes. The levels were normalized against the cell cytoplasmatic content.

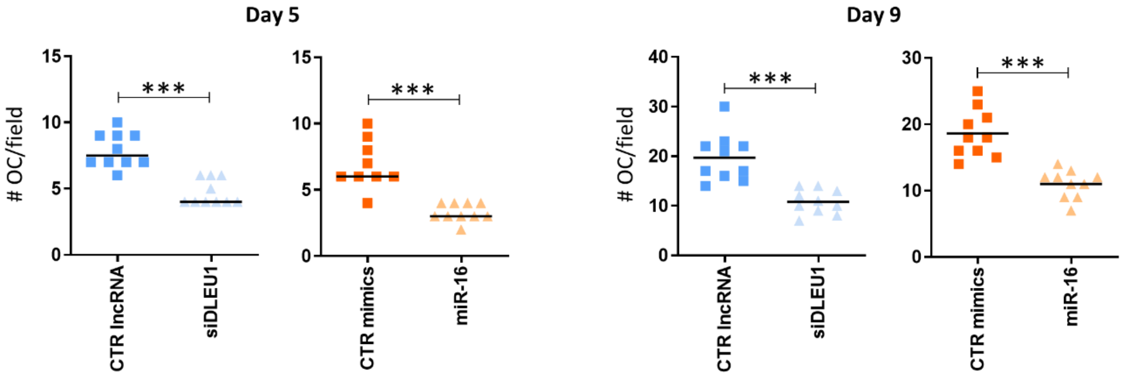

Supplementary Figure 5. Representative distribution of the number of OC per field of one donor at day 5 and 9 of the differentiation process. Each dot represents the mean of OCs per well (7 random fields were counted per well).

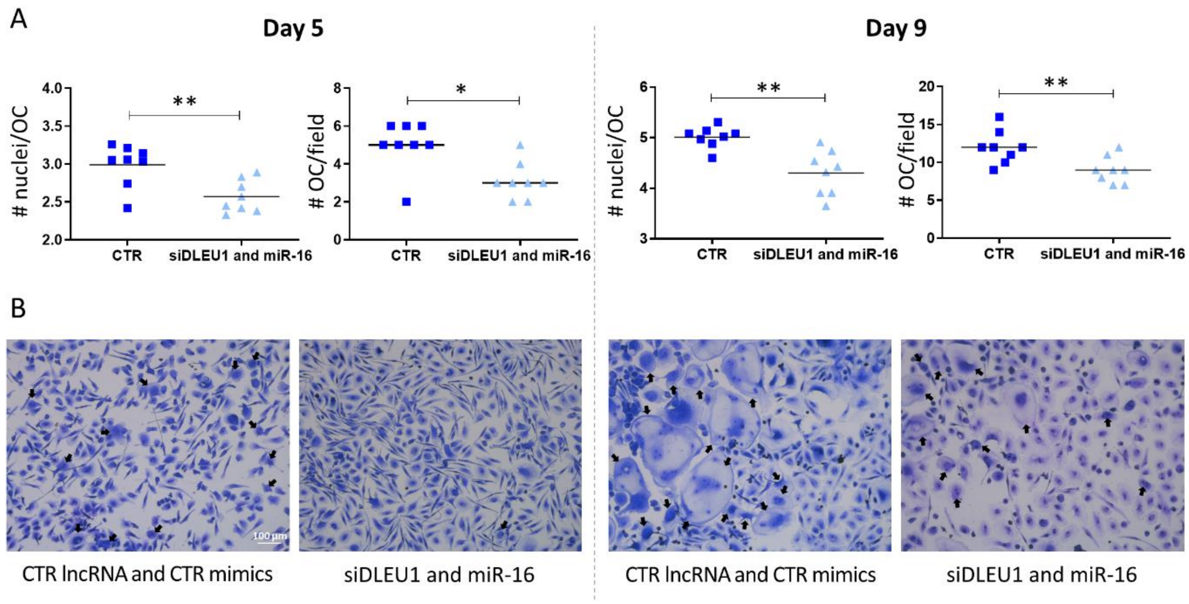

Supplementary Figure 6. Impact of the co-transfection of *DLEU1* and miR-16 on multinuclearity. A) Impact on the number of nuclei per OC (# nuclei / OC) and number of OCs per field (# OC / field) (*N*=3), at days 5 and 9. Each dot represents the mean of OCs from 7 random fields per each well. **B)** Representative images of OCs co-transfected with siDLEU1 and miR-16 at day 3 and left to differentiate until day 5 and 9. Black arrows are highlighting the multinucleated OCs.

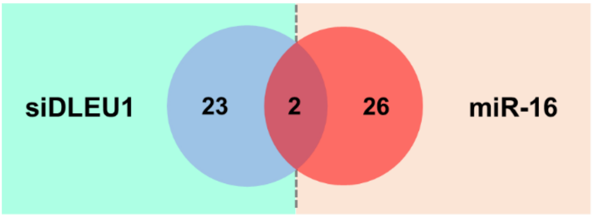

Supplementary Figure 7. Venn diagram showing the number of proteins differentially impacted by siDLEU1 and miR-16 mimics.

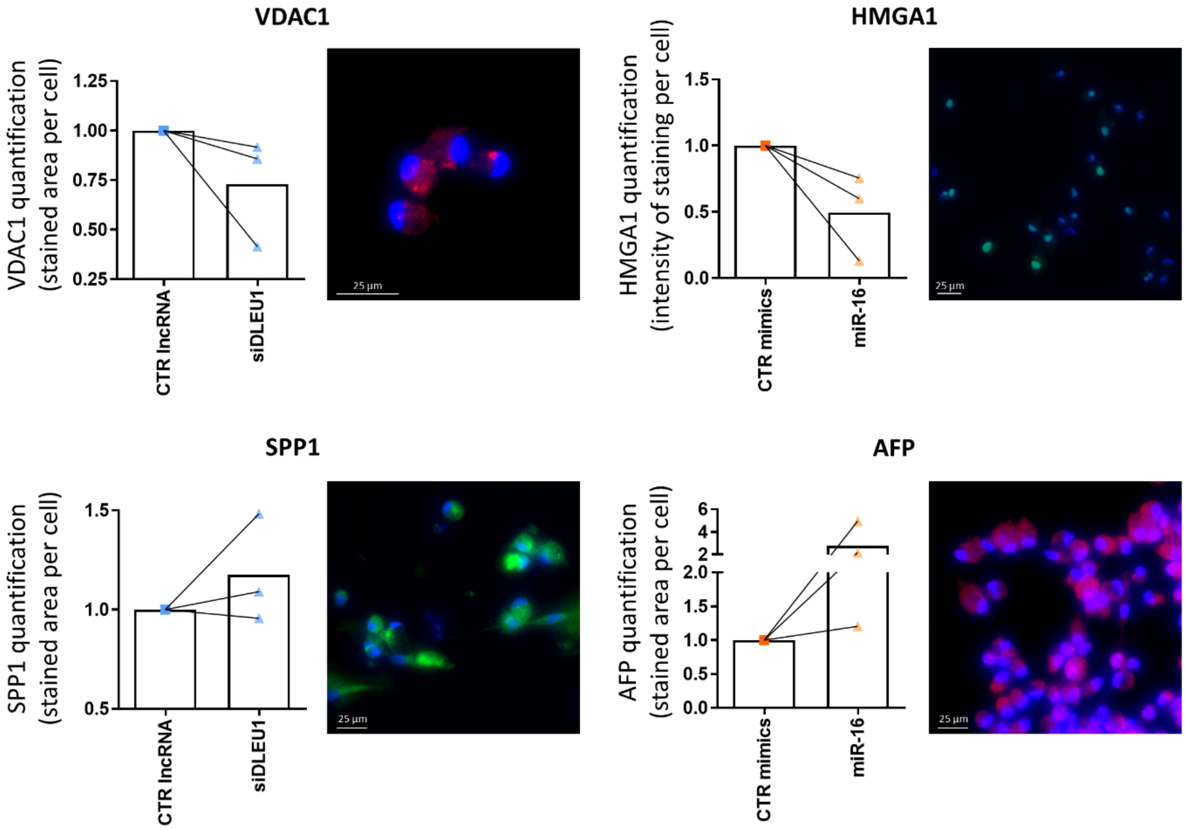

Supplementary Figure 8. Effect of siDLEU1 and miR-16 on the levels of proteins detected to be differentially expressed in the proteomic analysis. AFP, VDAC1 and SPP1 are represented as the area stained per cell normalized against the respective control, while HMGA1, found in the nucleus is represented as the intensity of staining per cell normalized against the respective control (*N*=3). Immunocytochemistry images are provided for each staining.

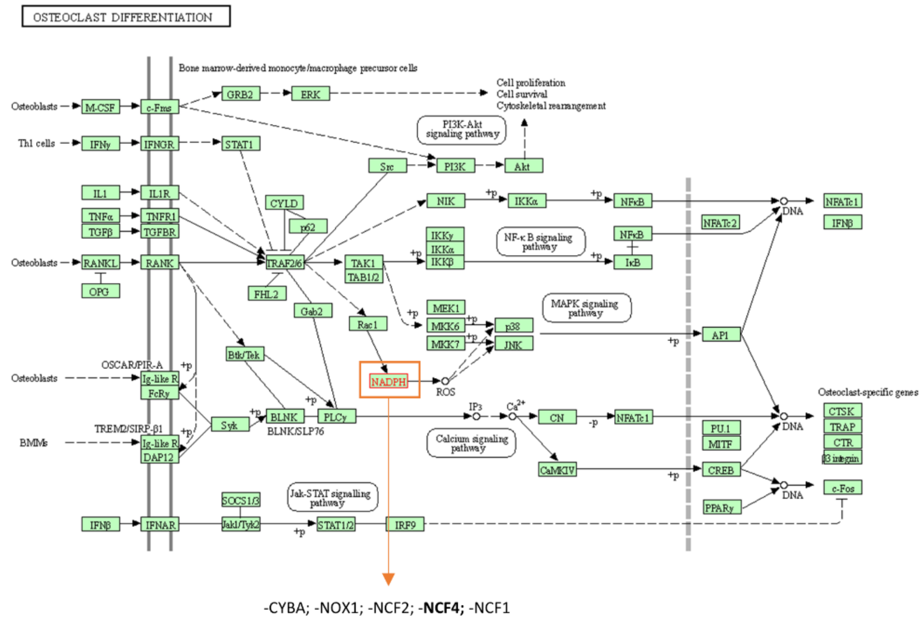

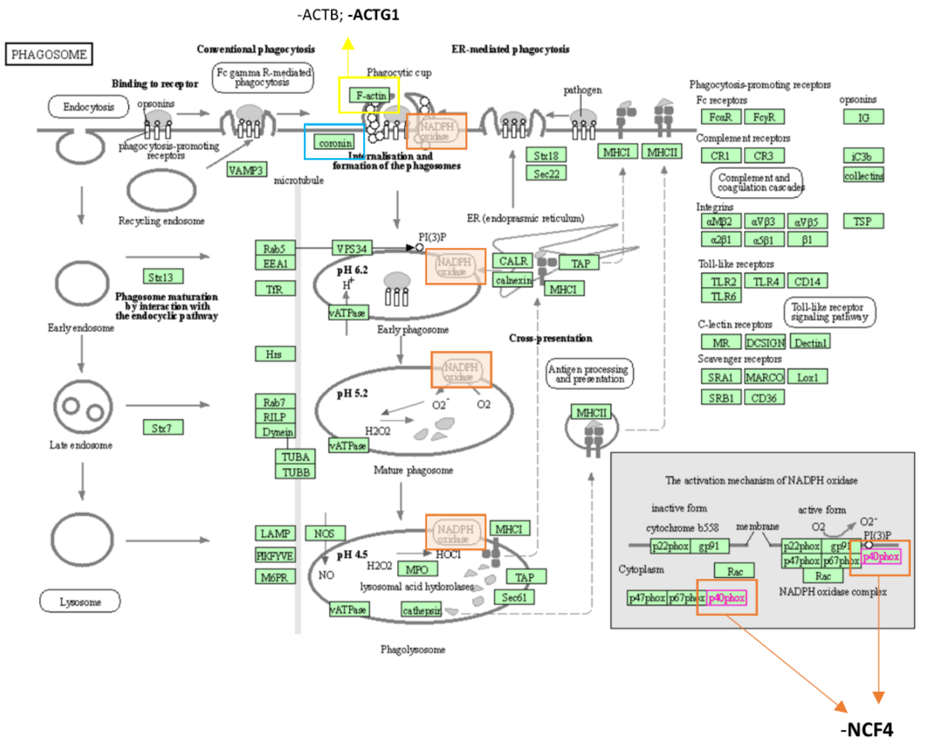

Supplementary Figure 9. “Osteoclast differentiation” and “phagosome” are KEGG pathways predicted to be impacted in response to *DLEU1* silencing. Proteins discovered to be differentially expressed following proteomic analysis are highlighted in bold.

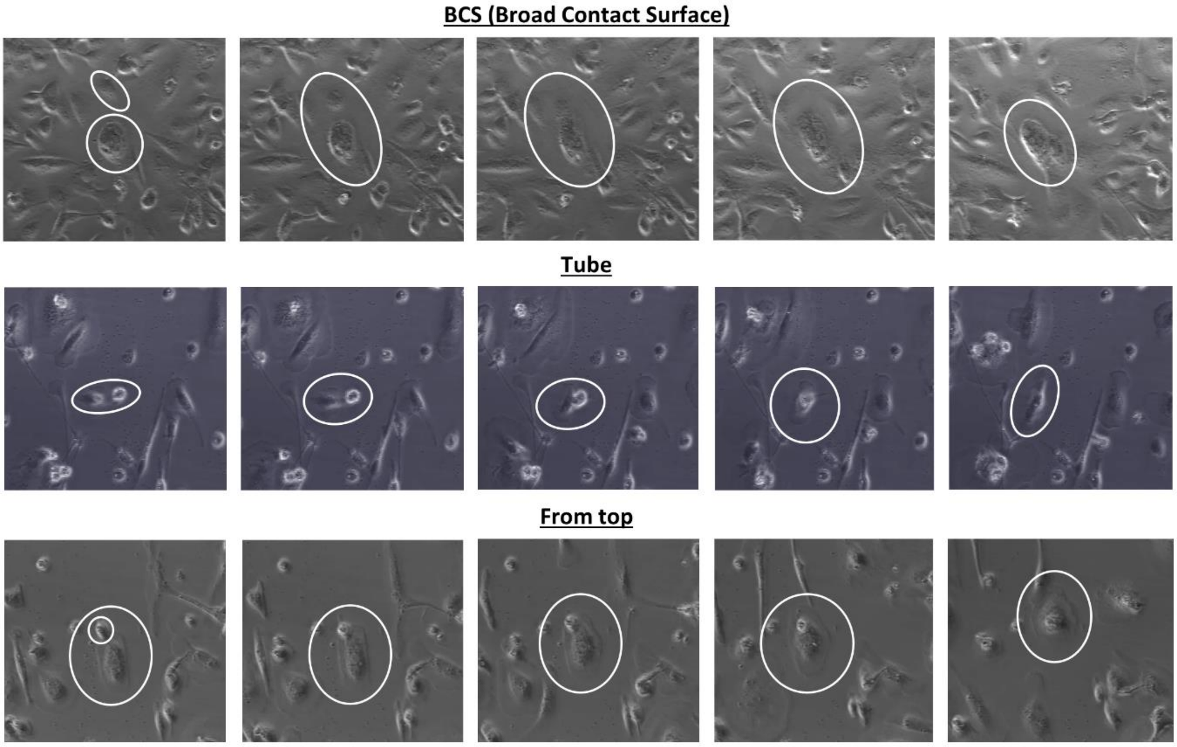

Supplementary Figure 10. Representative images of the different types of fusion modalities used in our analyses.

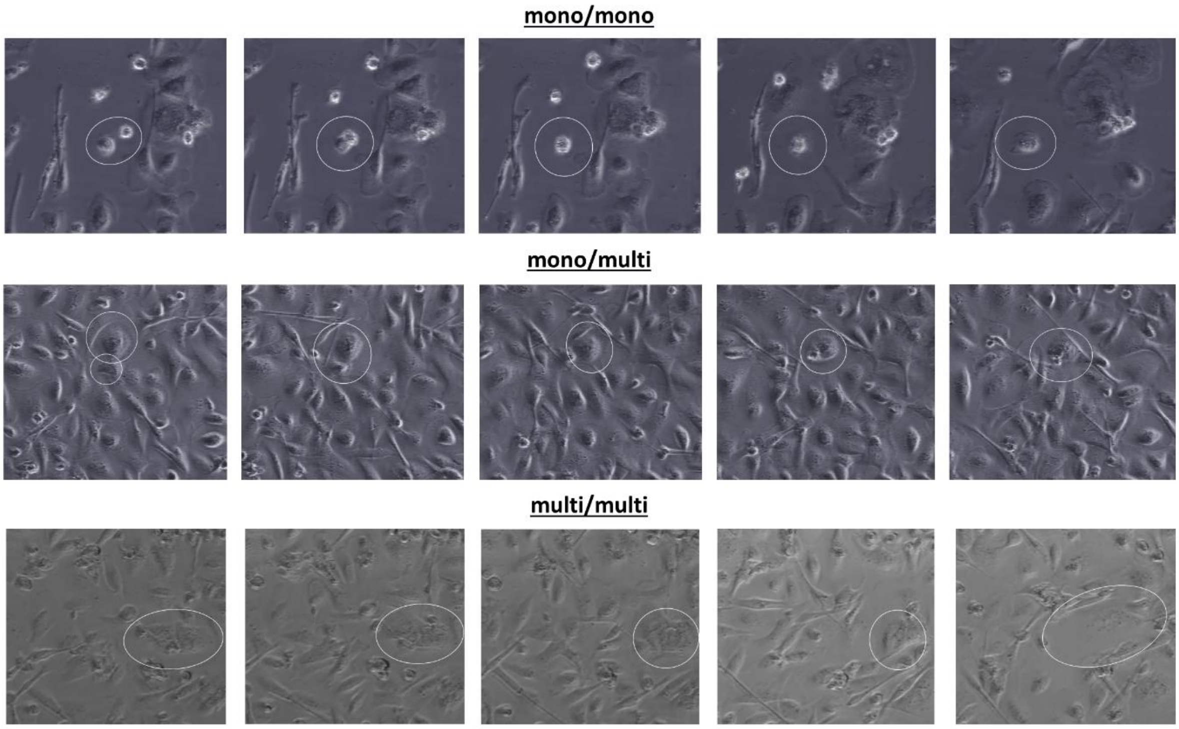

Supplementary Figure 11. Representative images of the different types of fusion pairs used in our analyses.

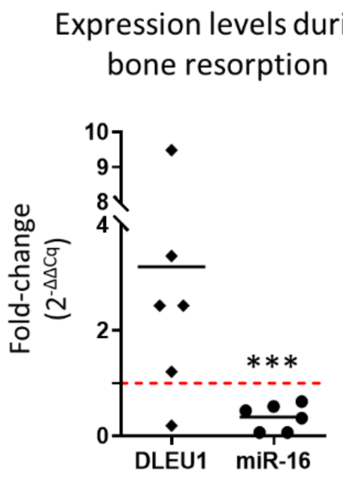

Supplementary Figure 12. Fold-change of expression levels of *DLEU1* and miR-16 during bone resorption. Mature OCs (non-transfected) were seeded onto bone slices at day 9 and left to resorb until day 12 (*N*=6). RNA was collected from OCs 24 h and 72 h after being seeded on top of bone slices and the results are presented relative to the 24 h timepoint.

Legend

Supplementary Video 1. Time-lapse video of osteoclasts performing pits and trenches.
