# Supplementary material for: Stage-specific modulation of multinucleation, fusion, and resorption by the long non-coding RNA DLEU1 and miR-16 in human primary osteoclasts
Source: Cell Death Dis. 2024 Oct 11;15(10):741. doi: 10.1038/s41419-024-06983-1 (PMC11467329; doi:10.1038/s41419-024-06983-1)
Supplement: Supplementary file 2 — Supplementary Tables [file 41419_2024_6983_MOESM2_ESM.pdf]

Supplementary files

Stage-Specific Modulation of Multinucleation, Fusion and Resorption by the Long Non-coding RNA DLEU1 and miR-16 in Human Primary Osteoclasts

Sara Reis Moura [1,2,3], Ana Beatriz Sousa [1,2,3], Jacob Bastholm Olesen [4,5,6], Mário Adolfo Barbosa [1,2], Kent Søe [4,5,6,*], Maria Inês Almeida [1,2,3,*,#]

* These authors contributed equally

Affiliations

1. i3S - Instituto de Investigação e Inovação em Saúde, Universidade do Porto, Porto, Portugal
2. INEB – Instituto de Engenharia Biomédica, Universidade do Porto, Portugal
3. ICBAS - Instituto de Ciências Biomédicas Abel Salazar, Universidade do Porto, Porto, Portugal
4. Department of Pathology, Odense University Hospital, Odense, Denmark
5. Clinical Cell Biology, Pathology Research Unit, Department of Clinical Research, University of Southern Denmark, Odense, Denmark
6. Department of Molecular Medicine, University of Southern Denmark, Odense, Denmark

#Corresponding author

Address: Rua Alfredo Allen, 208, 4200-135 Porto, Portugal

Email: ines.almeida@i3s.up.pt

Phone: +351 220 408 800

This file contains 2 Supplementary Tables.

Supplementary Table I. Mature miRNA sequences according to miRbase annotations (http://www.mirbase.org/).

miRNA	Acession	Mature sequence (5' – 3')
hsa-miR-15a-5p	MIMAT0000068	UAGCAGCACAUAAUGGUUUGUG
hsa-miR-16-5p	MIMAT0000069	UAGCAGCACGUAAAUAUUGGCG

Supplementary Table II. Primers used for reverse transcription quantitative real-time PCR.

Gene symbol	Name	Origin	Primer sequence (5' – 3')
ACP5	Tartrate resistant-acid phosphatase 5	Human	Fw: CGACCATTGTTAGCCACATACG
			Rv: TCGTCCTGAAGATACTGCAGGTT
ACTB	Actin Beta	Human	Fw: TGGCACCCAGCACAATGAA
			Rv: CTAAGTCATAGTCCGCCTAGAAGCA
CTSK	Cathepsin K	Human	Fw: ATATGTGGGCCAGGATGAAAGTT
			Rv: TCGTTCCCCACAGGAATCTCT
DLEU1	Deleted in lymphocytic leukemia 1	Human	Fw: CGTGCATTTAAAACCGCC
			Rv: TGTCTGCATTGTGACTCAATTC
DLEU2	Deleted in lymphocytic leukemia 2	Human	Fw: AATGAAGACCACAATAAAAGAGACC
			Rv: TTCCTTGCAGTACACCTTTCAA
